# Effects of Zinc supplementation on serum lipids: a systematic review and meta-analysis

**DOI:** 10.1186/s12986-015-0023-4

**Published:** 2015-08-04

**Authors:** Priyanga Ranasinghe, WS Wathurapatha, MH Ishara, R. Jayawardana, P. Galappatthy, P. Katulanda, GR Constantine

**Affiliations:** Department of Pharmacology, Faculty of Medicine, University of Colombo, Colombo, Sri Lanka; Ministry of Health Care and Nutrition, Colombo, Sri Lanka; Institute of Health and Biomedical Innovation, Queensland University of Technology, Brisbane, Queensland Australia; Diabetes Research Unit, Department of Clinical Medicine, Faculty of Medicine, University of Colombo, Colombo, Sri Lanka

## Abstract

Zinc is a mineral that plays a vital role in many biological processes and plays an important role in insulin action and carbohydrate metabolism. It may also have a protective role in the prevention of atherogenesis. Numerous studies have evaluated the effects of Zinc supplementation on serum lipids in humans and have demonstrated varying results. We systematically evaluated the literature and performed a meta-analysis on the effects of Zinc supplementation on serum lipids. A five staged comprehensive search of the literature was conducted in the following databases; PubMed, Web of Science and SciVerse Scopus for studies published before 31st December 2014. All controlled clinical trial in humans, that included a Zinc supplement intervention, either alone or in combination with other micronutrients and evaluated effects on serum lipids (total cholesterol [TC], triglycerides [TG], LDL cholesterol [LDL-c] and HDL cholesterol [HDL-c]). A meta-analysis of selected studies was performed using RevMan v5.3. The Jaded scale was used to assess the methodological quality of the trials included in the systematic review. A total of 24 studies were included in Meta analysis, which included a total of 33 Zinc interventions, in a total of 14,515 participants in the Zinc intervention or control group. The duration of Zinc supplementation ranged from 1 month to 7.5 years. The dose of elemental Zinc supplemented ranged from 15–240 mg/day. The pooled mean difference for TC between Zinc supplemented and placebo groups from random effects analysis was −10.92 mg/dl (95 % CI: −15.33, −6.52; p < 0.0001, I^2^ = 83 %), while for HDL cholesterol it was 2.12 mg/dl (95 % CI: −0.74, 4.98; p = 0.15, I^2^ = 83 %). The pooled mean difference for LDL-c between Zinc supplemented and placebo group from random effect analysis was −6.87 mg/dl (95 % CI: −11.16,-2.58; p < 0.001, I^2^ = 31) and for TG it was −10.92 mg/dl (95 % CI: −18.56, − 3.28; p < 0.01, I^2^ = 69 %). In conclusion, Zinc supplementation has favourable effects on plasma lipid parameters. Zinc supplementation significantly reduced total cholesterol, LDL cholesterol and triglycerides. Therefore it may have the potential to reduce the incidence of atherosclerosis related morbidity and mortality.

## Introduction

Zinc is a mineral that plays a vital role in many biological processes, such as enzyme action, cell membrane stabilization, gene expression and cell signaling [1]. It is required for structural and functional integrity of more than 2000 transcription factors and 300 enzymes; hence, almost all metabolic pathways are in some ways reliant on at least one Zinc requiring protein [2, 3]. Zinc also plays an important role in insulin action and carbohydrate metabolism [4]. Studies have shown that diabetes is accompanied by hypozincemia and hyperzincuria [5, 6]. In addition Zinc is also an integral part of key anti-oxidant enzymes and Zinc deficiency impairs their synthesis, resulting in increased oxidative stress [7].

Zinc deficiency is known to affect 1/3^rd^ of the world’s population [8]. It is estimated that Zinc deficiency is a major factor contributing to 1.4 % of deaths worldwide [8]. Zinc deficiency is more common in developing countries, and although severe deficiency is rare in developed countries, marginal deficiency is thought to be relatively common [9, 10]. Zinc deficiency is associated with many diseases, including malabsorption syndrome, chronic liver disease, chronic renal disease, sickle cell disease, diabetes and malignancy [11]. Animal studies have shown that Zinc deficiency has profound effects on the cell structure of the aorta, fatty acid metabolism and carbohydrate metabolism, being disadvantageous for maintaining vascular health [12]. Zinc deficiency renders vascular endothelial cells more susceptible to the effects of oxidative stress [13, 14]. Furthermore, in LDL receptor knock-out mice acute Zinc deficiency elicits changes in key transcription factors and adhesion molecules that are pro-atherogenic [15]. In human studies a strong negative association was observed between the dietary intake of Zinc and the incidence of diabetes and heart disease, as well as several of their associated risk factors including hypertension and hyper-triglyceridemia [16]. Hence Zinc may have a protective role in the prevention of atherogenesis [12].

Several human studies have demonstrated that Zinc supplementation reduces total cholesterol, LDL cholesterol and triglycerides, in addition to increasing the HDL cholesterol levels [17–20]. However, these results have been contradicted by other studies [21–23]. Even under the most rigorous study design conditions, a single well-planned study rarely provides definitive results [24]. Hence, changing clinical practices relying on a single high-profile clinical trial can be harmful to patients' health. Systematic reviews and meta-analyses on the other hand often have increased power and decreased bias as compared with the individual studies they include, and the careful pooling of treatment effects can provide the most accurate overall assessment of an intervention [24]. In 2008 Foster et al. performed a meta-analysis of controlled clinical trials to determine the effect of Zinc supplementation on serum lipids in humans [25]. They did not observe any beneficial effect of Zinc supplementation on plasma lipoproteins in the overall analysis, whilst in sub group analysis of healthy subjects Zinc supplementation was associated with a reduction in HDL cholesterol concentrations [25]. However, since then several recent studies have evaluated the effects of Zinc supplementation on serum lipids in humans and have demonstrated varying results [17, 21–23, 25–28]. Hence the present study aims to re-explore the area under discussion, by systematically evaluating the literature and performing an up to date meta-analysis on the effects of Zinc supplementation on serum lipids: total cholesterol (TC); LDL cholesterol (LDL-c); HDL cholesterol (HDL-c); and triglycerides (TG) in humans.

## Methods

The current systematic review was conducted in accordance with the PRISMA statement (Preferred Reporting Items for Systematic reviews and Meta-Analyses) for systematic reviews of interventional studies [29].

### Literature search

A five staged comprehensive search of the literature was conducted in the following databases; PubMed® (U.S. National Library of Medicine, USA), Web of Science® [v.5.4] (Thomson Reuters, USA) and SciVerse Scopus® (Elsevier Properties S.A, USA) for studies published before 31^st^ December 2014. During the first stage the above databases were searched using the following keywords; (‘Zinc’ or ‘Zn’ or ‘Zinc supplementation’ or ‘Zn supplementation’ or ‘Zinc therapy’ or ‘Zn therapy’) AND (‘Lipid(s)’or ‘Cholesterol’ or ‘LDL’ or ‘HDL’ or ‘Lipoprotein(s)’ or ‘Triglyceride(s)’).

In the second stage the total hits from the 3 databases were pooled and duplicates were removed. This was followed by screening of the retrieved articles by reading the article ‘title’ in the third stage and ‘abstracts’ in stage four. In the fifth stage individual manuscripts were screened, and those not satisfying inclusion criteria were excluded. To obtain additional data a manual search of the reference lists of articles selected in stage five was performed. This search process was conducted independently by two reviewers (PR and RJ) and the final group of articles to be included in the review was determined after an iterative consensus process.

### Inclusion and exclusion criteria

A study was considered eligible for data extraction if it was a controlled clinical trial in humans, that included a Zinc supplement intervention, either alone or in combination with other micronutrients and evaluated at least one of the following outcomes: TG,TC, LDL-c and HDL-c. Results were limited to studies conducted in humans, published in English, while conference proceedings, editorials, commentaries and book chapters/book reviews were excluded.

### Data extraction and analysis

A meta-analysis of selected studies examining the effects of Zinc supplementation on serum lipid parameters was performed using the Rev Man version 5.3 (Review Manager, Copenhagen: The Nordic Cochrane Centre, The Cochrane Collaboration, 2011) statistical software package. A random effect analysis was conducted for all comparisons and in all analyses a *p*-value < 0.05 was considered statistically significant. Forest plots were used to illustrate the study findings and meta-analysis results. Statistical heterogeneity was assessed using the χ^2^ test on Cochrane’s *Q* statistic [30] and by calculating *I*^*2*^ [31] and is considered significant if p < 0.05. TC, LDL-c, HDL-c and TG are reported as mg/dl, where studies reported as mmol/l a numerical conversion to mg/dl was done as follows; For TC, HDL-c, and LDL-c cholesterol, values presented in mmol/l multiplied by conversion factor 38.67 and for TG, conversion factor used was 88.57 [32]. In a study where there was several interventions including interventions with multiple supplements, the interventions using Zinc supplementation alone was used in the meta analysis as the elemental dose of Zinc was similar in all interventions [33]. In a study done by Farvid et al. intervention with least number of additional supplements was used to compare with the placebo group [34]. In cross-over studies the pooled estimate of Zinc and placebo groups after completion of entire cross-over scheme was used in the analysis [17, 19, 35]. Three separate sub-group analyses were performed; a) for group of studies using Zinc supplementation alone [17–19, 21–23, 26, 27, 33, 35–43], b) for group of studies done on healthy participants [35–37, 39–41, 44] and c) for group of studies done on non-healthy participants [17–23, 26–28, 33–35, 38, 42, 43, 45, 46].

### Quality assessment

The Jaded scale was used to assess the methodological quality of the trials included in the systematic review [47]. Each study was scored from 0 (‘poor’ quality) to 5 (‘good’ quality) according to the following criteria: 1) was the study described as randomized? ;2) was the study described as double blind?; 3) was there a description of withdrawals and dropouts? 3) was the method of randomization described in the paper and appropriate?; 4) was the method of blinding described and appropriate?. Each question would score a single point if the answer is ‘yes’ or zero points if the answer is ‘no’. Questions 4) and 5) would score −1 mark each if method of randomization was described, but inappropriate and method of blinding was described, but inappropriate respectively.

## Results

### Literature search

**Fig. 1:**
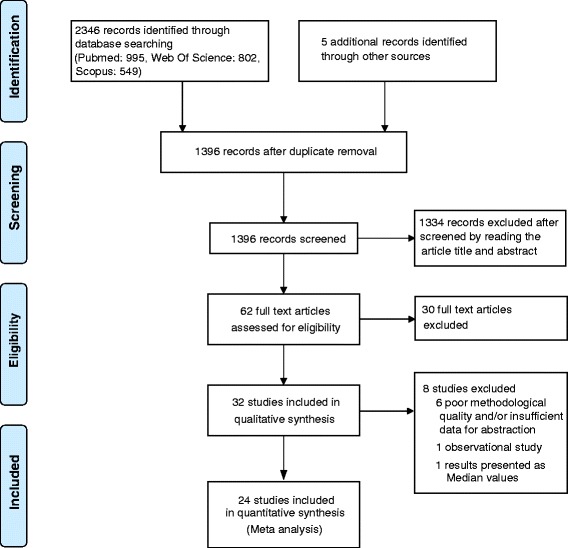
Summarized search strategy

Literature search was done according to the above search criteria and the search strategy is summarized in Fig. 1. The number of articles identified initially from the different databases were as follows; PubMed (n = 995), Web of Science (n = 802), SciVerse Scopus (n = 549). Five additional articles were identified by manual searching the reference lists of included studies. After removal of duplicates and screening of studies by reading the title, abstract and finally the full text, 32 studies were eligible to be included in systematic review. Descriptions of included studies are presented in Table 1. Only 24 studies were included in Meta analysis and the reasons for exclusion of 8 studies [48–55] are also mentioned in Fig. 1.

**Table 1:** Description of included studies

Authors^[ref]^ Year of Publication; Country	Study design	Duration of Zn supplementation	*n* control(s)/placebo, *n* Zn supplement(s) *n* Other supplements	Gender	Age	Health status	Formulation Elemental Zn dose(s)	Lipid parameters studied	Significant outcomes
Afkhami-Ardekani et al. [18] 2008; Iran	R, P	1.5 months	20 20	Both	52.67 ± 8.6	Type-2 diabetes patients	ZnSO_4_ 660 mg/day^a^	TG, TC, HDL-c, LDL-c	Reduction in TG, TC and LDL
Age-Related Eye Disease Study Research Group [48] 2002; United States	R, DB, P	5 years	166 202 (Zn),168(Zn + antioxidants) 181 (antioxidants)	Both	55-80	Patients with Age-related macular degeneration	ZnO 80 mg/day	TG, TC, HDL-c, LDL-c	Lipid profile not significantly affected by long-term supplementation with Zinc
Black et al. [36] 1988; United States	R, DB, P	3 months	9 13,9	Males	19-29	Healthy	Zn gluconate 50 mg/day (n = 13) 75 mg/day (n = 9)	TG, TC, HDL-c, LDL-c, VLDL	Serum TC, VLDL, LDL-c, TG not affected. Both Zn groups had significantly lower HDL than placebo group and lower than baseline
Bogden et al. [37] 1988; United States	R, DB, P	3 months	36 36, 31	Both	60-89	Healthy	Zn acetate 15 mg/day (n = 36) 100 mg/day (n = 31)	TC, HLD-c,	Serum TC and HDL-c was not altered significantly by Zinc
Boukaïba et al. [35] 1993; France	R, DB, C	2 months	23, 21	Both	73-106	Healthy 2 groups Reference- BMI >24 kg/m^2^ (n = 23)	Zn gluconate 20 mg/day	TG, TC, HDL-c, LDL-c	Reduced TC in both Lean and Reference groups than placebo. Reduced HDL in Reference group. Higher TG in Lean group. No effect on TG in Reference group. Ratio of LDL-c to HDL-c not affected.
			23, 21						
						Lean – BMI < 21 kg/m^2^ (n = 21)			
Brewer et al. [49] 1991; United States	O	1-5 years	No controls	Both	NM	Wilson’s disease	NM	TG, TC, HDL-c,	HDL level reduced in males only. TC reduced in both genders.
								LDL-c, TC/HDL-c	
			11(F), 13(M)						
Chevalier et al. [38] 2002; United States	R, DB, P	3 months	10	Both	23-80	End-Stage Renal	ZnSO_4_	TC, HDL-c,	TC and LDL-c increased. No change in HDL-c
						Disease on Haemodialysis	50 mg/day	LDL-c	
			10						
Crouse et al. [39] 1984; United States	R, DB, P	2 months	10,11	Males	20-55	Healthy	ZnSO_4_	TG, TC, HDL-c,	No significant change in lipid parameters in both groups
						2 groups	28.7 mg/day	LDL-c	
			11,12			Endurance trained			
						(n = 21)			
						Sedentary (n = 23)			
Farvid et al. [34] 2004; Iran	R, DB, P	3 months	18	Both	30-69	Type-2	ZnSO_4_	TG, TC, HDL-c,	Co-supplementation of Mg, Zn, Vitamins C and E significantly increases HDL-c. TG, TC, LDL-c not altered
						Diabetes patients	30 mg/day	LDL-c	
			16 (Zn + Mg), 17(Zn + Mg + Vit.C + Vit.E) 18 (Vit C+ Vit.E)						
Federico et al. [45] 2001; Italy	R, CC	2 months	30	Both	46-61	Gut cancer patients	NM	TC	No Significant reduction in TC
							21 mg/day		
			30 (Zn + Se)						
Feillet-Coudray et al. [40] 2006; France	R, DB, P	6 months	16 (F),16 (M), 16 (F), 16 (M)	Both	55-70	Healthy	Zn gluconate	TG, TC, HDL-c,	No significant change in lipid parameters
			16 (F), 16 (M), 15 (F), 16 (M)						
								LDL-c	
							15 mg/day(n = 32)		
							30 mg/day(n = 31)		
Foster et al. [33] 2013; Australia	R, DB, P	3 months	10	Females	65.0 ± 7.8	Type-2	Zn sulphate	TG, TC, HDL-c,	No significant change in lipid parameters
						Diabetes patients	40 mg/day	LDL-c	
			12 (Zn), 11 (Zn + ALA)						
			10 (ALA)						
Freeland-Graves et al. [50] 1982; United States	R, DB, P	2 months	8	Females	18-40	Healthy	Zn Acetate	HDL-c	A transient non-dose related reduction in HDL-c
							15 mg/day (n = 8)		
			8, 8, 8				50 mg/day (n = 8)		
							100 mg/day (n = 8)		
Gatto and Samman [51] 1995; Australia	R, B, C	1 month	10	Males	24.3 ± 4.2	Healthy	ZnSO_4_	TG, TC,	No significant changes in TC, LDL-c or TG. HDL_2_:HDL_3_ ratio increased
			10				50 mg/day	HDL-c,	
								LDL-c	
Gunasekara et al. [46] 2011; Sri Lanka	R, B, P	4 months	32	Both	M-54.6 ± 7.0	Type-2	ZnSO4	TG, TC, HDL-c,	Reduced TC, LDL and TC/HDL-c ratio only in the group receiving Zinc + MVM supplementation.
					F- 54.9 ± 9.0	Diabetes patients	22 mg/day	LDL-c	
			28 (Zn + MVM)						
			26 (MVM)						
Hashemipour et al. [17] 2009; Iran	R, DB, C	2 months	60	Both	6-10	Obese	ZnSO4	TG, TC, HDL-c,	TC, LDL-c and TG reduced. No change in HDL-c
							20 mg/day	LDL-c	
			60						
Hercberg et al. [44] 2005; France	R, DB, P	7.5 years	3869 (F), 2508(M)	Both	35-60	Healthy	Multi-vitamin	TG, TC, HDL-c,	Significant reduction of HDL-c in men. No significant change in other lipid parameters
			3844 (F), 2520(M)				(Zn 20 mg/day)	LDL-c	
Hininger-Favier et al. [41] 2007; Europe	R, DB, P	6 months	130	Both	55-85	Healthy	Zn Gluconate	TG, TC, HDL-c,	No effect with Zinc 15 mg/day or 30 mg/day
							15 mg/day	LDL-c, LDL:HDL	
			126, 131				(n = 126)		
							30 mg/day (n = 131)		
Hooper et al. [52] 1980, United States	P	5 weeks	8	Males	23-35	Healthy	ZnSO4	TG, TC, HDL-c,	25 % reduction of HDL-c. TC, TG, and LDL-c no significant change.
							160 mg/day		
								LDL-c	
			12						
Kadhim et al. [20] 2006; Iraq	DB, P	3 months	15 (Metformin + Placebo)	Both	40-64	Type-2 Diabetes patients	Zn acetate	TG, TC, HDL-c,	Reduced TC, TG and LDL-c. HDL-c significantly increased.
			18 (Metformin + Melatonin + Zn)				50 mg/day^a^	LDL-c	
			13 (Melatonin + Zn)						
Khan et al. [42], 2013; India	R, P	3 months	21	Both	40-69	Type-2 Diabetes patients	ZnSO4	TG, TC, HDL-c,	Decrease in TG. HDL-c increased. No change in TC and LDL-c.
			23				50 mg/day	LDL-c	
Kim and Lee [21] 2012; South Korea	P	2 months	20	Females	19-28	Obese	Zn gluconate	TG, TC, HDL-c	No significant change in lipid parameters
							30 mg/day		
			20						
Li et al. [28] 2010; China	R, DB, P	6.5 months	29	Females	18-55	Obese	Multi-vitamin	TG, TC, HDL-c,	Reduced TC and LDL-c and increased HDL-c in multivitamin & mineral supplemented group. No change in TG.
							(Zn 15 mg/day)	LDL-c	
			30 (multivitamin & mineral)						
			28 (Calcium)						
Partida-Hernández et al. [19] 2006, Mexico	R, DB, C	3 months	27	Males	35-65	Type-2	ZnSO4	TG, TC, HDL-c,	Reduced TC, TG, VLDL-c. Increased HDL-c
						Diabetes patients	100 mg/day^a^	LDL-c, VLDL-c	
			27						
Payahoo et al. [26] 2013; Iran	R, DB, P	1 month	30	Both	18-45	Obese	Zn gluconate	TG, TC, HDL-c,	TG level decreased.
			30						
							30 mg/day^a^	LDL-c	No change in other lipid parameters
Rahimi-Ardabili et al. [27] 2012; Iran	R, DB, P	2 months	30	Both	52.8 ± 12.7	End-Stage Renal	ZnSO4	TG, TC, HDL-c,	No change in TC, TG and LDL-c.
			30						
						Disease on Haemodialysis	100 mg/day^a^	LDL-c	Increase in HDL
RangaRao et al. [53] 1990; India	CC	1 month	5	Males	NM	Type-1	ZnSO4	TG, TC, HDL-c	No significant change in lipid parameters
						Diabetes patients	660 mg/day^a^		
			7						
Roozbeh et al. [22] 2009; Iran	R, DB, P	1.5 months	26	Both	55.7	End-Stage Renal	ZnSO4	TG, TC, HDL-c,	Increase in TC, TG, LDL-c and HDL-c
						Disease on Haemodialysis	50 mg/day	LDL-c	
			27						
Samman and Roberts [54] 1988; Australia	R, DB, C	1.5 months	41	Both	M-28.2 ± 2.0	Healthy	ZnSO4	TC, LDL-	No change in TC. In females LDL-C reduced. HDL2 increased and HDL 3 decreased.
			41		F- 26.8 ± 1.6		150 mg/day	c, HDL-c,	
								HDL2, HDL3	
Seet et al. [23] 2011; Singapore	R, B, P	3 months	20	Males	NM	Type-2	Zn gluconate	TG, TC, HDL-c,	No significant change in lipid parameters
						Diabetes patients	240 mg/day	LDL-c	
			20						
Shah et al. [43] 1988; India	R, P	1 month	10	Males	31-70	Ischaemic Heart	ZnSO4	TC, TG, α- and	Significant reduction in TC, β-lipoprotein. Increase in α-lipoprotein. No change in TG
						Disease patients	600 mg/day^a^	β-lipoprotein	
			10						
Thurnham et al. [55] 1988; Chiina	R, DB, P	13.5 months	610^b^	Both	35-64	Healthy	Zn gluconate	TC	No significant change
							50 mg/d		

*ALA*-α-linolenic acid, *B*-Single blinded, C-Cross over; *CC*-Case–control, *DB*-Double Blinded, *F*-female, *HDL-c*-High Density Lipoprotein-Cholesterol, *LDL-c*-Low Density Lipoprotein-Cholesterol, *M*-male, *MVM*- multivitamin/mineral,*NM*-Not Mentioned, *O*- Observational, *P*-Parallel, *R*–Randomized, *TC*-Total Cholesterol, *TG*-Triglycerides, Age presented as mean ± SD in years where data were available and as age range in other studies

^a^Dosage of formulation,; ^b^Number of participants in each group is not mentioned

### Description of the studies and Quality assessment

Studies that were included in the meta-analysis are English-language, human, controlled trials. Out of the total of 33 Zinc interventions included in meta-analysis, 26 interventions [17–19, 21–23, 26, 27, 33, 35–43] investigated the effects of Zinc supplementation alone on plasma lipids while other 7 interventions investigated the effect of supplementation of Zinc together with other vitamins and/or minerals. Duration of Zinc supplementation ranged from 1 month to 6.5 months with the exception of one long term study in which Zinc was supplemented for 7.5 years [44]. The dose of elemental Zinc supplemented in these interventions ranged from 15–240 mg/day (average dose of elemental Zinc per intervention: 39.3 mg/day). A variety of Zinc anions were used, including sulfate [17–19, 22, 27, 33, 34, 38, 39, 42, 43, 46], gluconate [21, 23, 26, 35, 36, 40, 41] and acetate [20, 37] or undefined [28, 44, 45].

In total, 14,515 participants were assigned to a Zinc intervention or control group. The age range of participants was 19–106 years except one study which was done in children aged 6–10 years. Out of 24 studies, 7 studies (16 interventions) involved healthy participants. Of the remaining trials, 8 studies were undertaken in those with type 2 Diabetes, 4 studies in obese individuals, 3 studies in subjects with end stage renal failure undergoing haemodialysis, 1 study in gut cancer patients and 1 study in patients with Ischemic heart disease. The mean jaded scale score for all trials included in meta-analysis was 3.13, out of a maximum score of 5 and 16 out of 24 studies scored ≥ 3 marks (Table 2). Two studies that scored zero points were excluded from the meta-analysis due to poor methodological quality (Fig. 1) [49, 52].

**Table 2:** Jaded scale

Study	Randomised	Double blind	Withdrawals and drop outs	Randomisationmethod described and appropriate	Blinding method described and appropriate	Total
Afkhami-Ardekani et al., 2008	1	0	1	0	N/A	2
Age-Related Eye Disease Study Research Group, 2002	1	1	0	0	0	2
Black et al., 1988	1	1	1	0	0	3
Bogden et al., 1988	1	1	0	1	0	3
Boukaïba et al., 1993	1	1	1	0	0	3
Brewer et al., 1991	0	0	0	0	0	0
Chevalier et al., 2002	1	1	1	0	0	3
Crouse et al., 1984	1	1	1	0	0	3
Farvid et al., 2004	1	1	1	1	0	4
Federico at al., 2001	1	0	1	0	N/A	2
Feillet-Coudray et al., 2006	1	1	1	0	0	3
Foster et al., 2013	1	1	1	1	1	5
Freeland-Graves et al., 1982	1	1	0	0	0	2
Gatto et al., 1995	1	0	1	0	N/A	2
Gunasekara et al., 2011	1	0	1	1	0	3
Hashemipour et al., 2009	1	1	1	1	0	4
Hercberg et al., 2005	1	1	1	1	1	5
Hininger-Favier et al., 2007	1	1	1	1	1	5
Hooper et al., 1980	0	0	0	0	0	0
Kadhim et al., 2006	0	1	1	N/A	0	2
Khan et al., 2013	1	0	1	0	N/A	2
Kim et al., 2012	0	0	1	0	0	1
Li et al., 2010	1	1	1	1	0	4
Partida-Hernández et al., 2006	1	1	1	0	0	3
Payahoo et al., 2013	1	1	1	1	1	5
Rahimi-Ardabili et al., 2012	1	1	0	0	0	2
RangaRao et al., 1990	0	0	1	0	0	1
Roozbeh et al., 2009	1	1	1	0	1	4
Samman et al., 1988	1	1	1	0	0	3
Seet et al., 2011	1	0	1	0	N/A	2
Shah et al., 1988	1	0	1	0	N/A	2
Thurnham et al., 1988	1	1	0	0	0	2

### Effect Zinc supplementation on total cholesterol

**Fig. 2:**
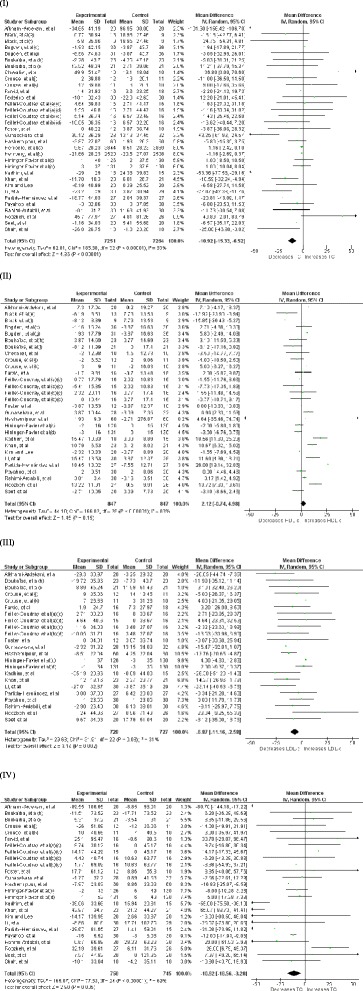
Forest plots showing effect of Zinc supplementation on; (**I**) Total cholesterol, (**II**) HDL cholesterol, (**III**) LDL cholesterol, (**IV**) Triglycerides. a- female, (b)- male, (c)- Zinc supplementation 15 mg/day, (d)- Zinc supplementation 30 mg/day, (e)- Zinc supplementation 50 mg/day, (f)- Zinc supplementation 75 mg/day, (g)- Zinc supplementation 100 mg/day, (h)- reference group, (i)- Lean group, (j)- Sedentary males, (k)- Trained males

**Fig. 3:**
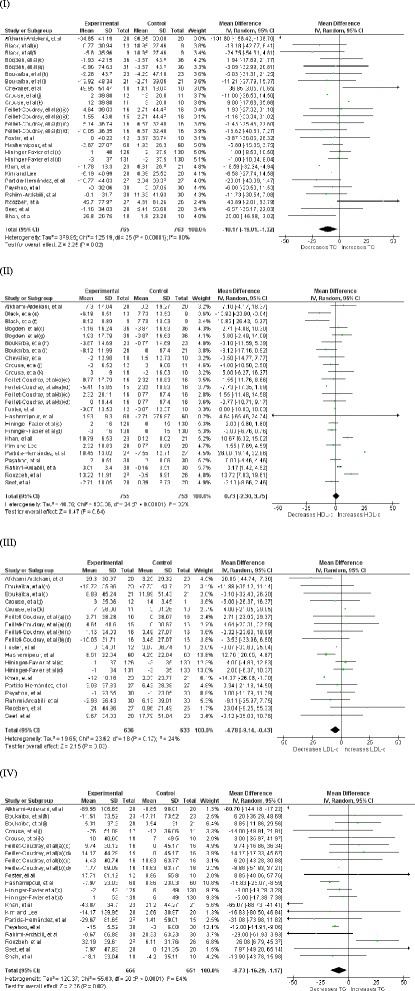
Forest plots showing effect of Zinc supplementation alone (sub-analysis) on; (**I**) Total cholesterol, (**II**) HDL cholesterol, (**III**) LDL cholesterol, **IV** Triglycerides. (a)- female, (b)- male, (c)- Zinc supplementation 15 mg/day, (d)- Zinc supplementation 30 mg/day, (e)- Zinc supplementation 50 mg/day, (f)- Zinc supplementation 75 mg/day, (g)- Zinc supplementation 100 mg/day, (h)- reference group, (i)- Lean group, (j)- Sedentary males, (k)- Trained males

**Fig. 4:**
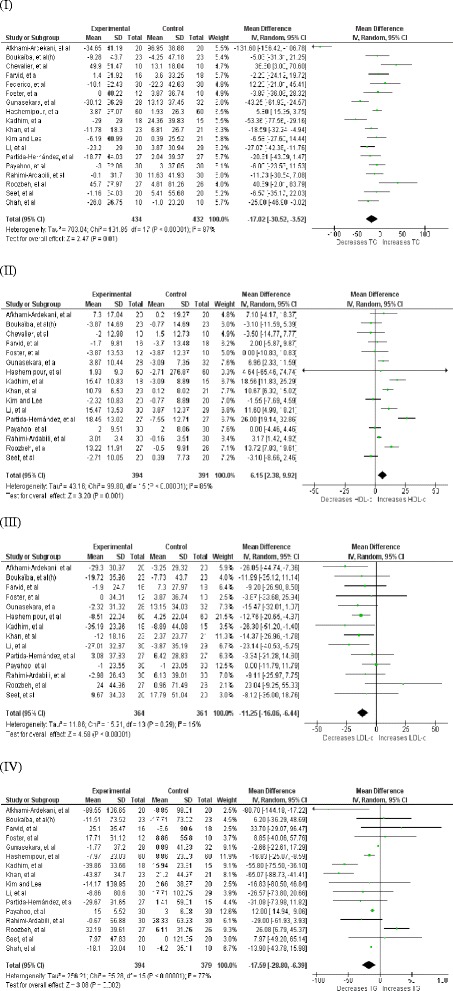
Forest plots showing effect of Zinc supplementation in non-healthy participants on; (**I**) Total cholesterol, (**II**) HDL cholesterol, (**III**) LDL cholesterol, (**IV**) Triglycerides

**Fig. 5:**
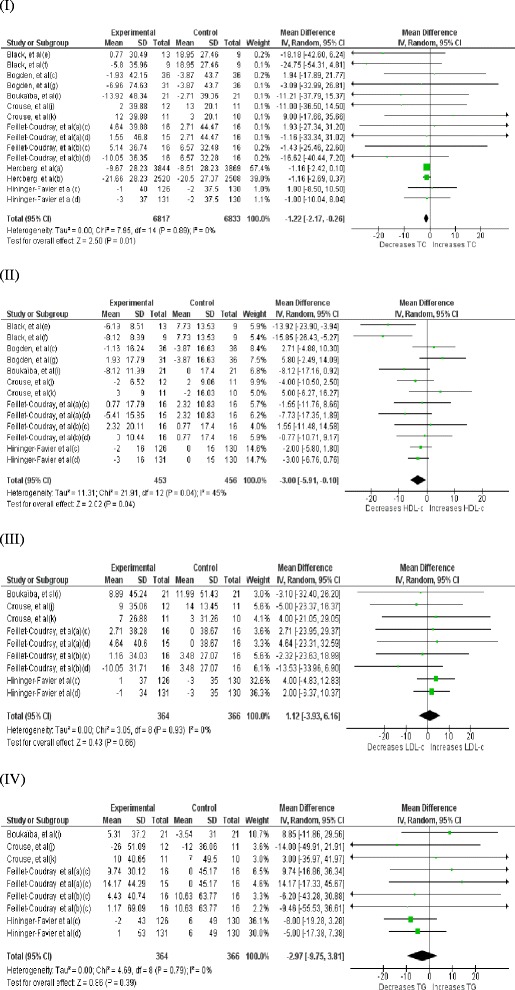
Forest plots showing effect of Zinc supplementation in healthy participants on; (**I**) Total cholesterol, (**II**) HDL cholesterol, (**III**) LDL cholesterol, (**IV**) Triglycerides

Effect of Zinc supplementation on total cholesterol concentration was studied in all 24 studies (33 interventions, n = 14515) [17–23, 26–28, 33–46] included in the meta-analysis. There was a statistically significant reduction in TC concentration in the Zinc supplemented group. The pooled mean difference for TC between Zinc supplemented and placebo groups from random effects analysis was −10.92 mg/dl (95 % CI: −15.33, − 6.52; p < 0.0001). However statistical heterogeneity as indicated by I^2^ = 83 % (p < 0.05) of the data prevents the evaluation of a pooled estimate for TC (Fig. 2 (I)). In the subgroup-analyses, the group of interventions (26 interventions, n = 1528) in which Zinc was supplemented alone demonstrated a similar, statistically significant reduction in TC concentration in comparison to control groups. The pooled mean difference for TC between Zinc supplemented and placebo groups from random effect analysis was −10.72 mg/dl (95 % CI: −19.01, −1.32; p <0.05) (Fig. 3(I)) and statistical heterogeneity as indicated by I^2^ = 80 % (p < 0.05). When studies were grouped by health status, reduction in TC in comparison to control groups was statistically significant and was greater in magnitude (−17.02 mg/dl [95 % CI: −30.52, −3.52; p < 0.05], [I^2^ = 87, p < 0.05]) among non-healthy participants (18 interventions, n = 866) (Fig. 4 (I)). Zinc supplementation among healthy participants (15 interventions, n = 13,650) demonstrated minor but statistically significant reduction in TC (−1.22 mg/dl [95 % CI: −2.17, −0.26; p < 0.05], [I^2^ = 0, p >0.05] ) (Fig. 5(I)).

### Effect of Zinc supplementation on HDL cholesterol

Twenty one studies (29 interventions, n = 1,694) [17–23, 26–28, 33–42, 46] explored the effect of Zinc supplementation on HDL cholesterol. The forest plot for overall analysis of HDL cholesterol (Fig. 2(II)) shows the pooled mean difference for HDL cholesterol between Zinc supplemented and placebo groups from random effect analysis was 2.12 mg/dl (95 % CI: −0.74, 4.98; p = 0.15) and statistical heterogeneity as indicated by I^2^ = 83 % (p < 0.05). Also the group of interventions (25 interventions, n = 1,508) employing Zinc supplementation alone did not demonstrate a statistically significant increase in HDL-c levels (+0.73 mg/dl, 95 % CI: −2.30, 3.75, p = 0.64) in subgroup-analysis (Fig. 3(II)). However, as Fig. 4(II) illustrates Zinc supplementation among non-healthy participants (16 interventions, n = 785) demonstrated a considerable and statistically significant increase in HDL-c (+6.15 mg/dl [95 % CI: 2.38, 9.92; p < 0.05], [I^2^ = 85, p < 0.05]). In contrast to this Zinc supplementation in healthy participants (13 interventions, n = 909) demonstrated a significant reduction in HDL-c (−3 mg/dl [95 % CI: −5.91, 0.10; p < 0.05], [I^2^ = 45, p < 0.05]) (Fig. 5 (II)).

### Effect of Zinc supplementation on LDL cholesterol

There were 17 studies [17–20, 22, 23, 26–28, 33–35, 39–42, 46] (23 interventions, n = 1,455) in which the effect of Zinc supplementation on LDL cholesterol was studied. Forest plot for LDL-c (Fig. 2(III)) shows there is a statistically significant reduction in LDL-c in Zinc supplemented group. The pooled mean difference for LDL-c between Zinc supplemented and placebo group from random effect analysis was −6.87 mg/dl (95 % CI: −11.16, −2.58; p < 0.001) and the statistical heterogeneity of the data as indicated by I^2^ = 31 was insignificant (p = 0.08). Forest plot for subgroup analysis (Fig. 3(III)) of LDL-c shows the pooled mean difference for LDL-c between Zinc alone supplemented group and placebo groups from random effect analysis was −4.78 mg/dl (95 % CI: −9.14, −0.43; p < 0.05) and the statistically heterogeneity was I^2^ = 24 (p = 0.17). When the interventions done in non- healthy participants (14 interventions, n = 725) were grouped together, Zinc supplementation demonstrated a significant reduction in LDL-c (−11.25 mg/dl [95 % CI: −16.06, −6.44; p < 0.05], [I^2^ = 15, p > 0.05]) and the magnitude of reduction was greater than that in overall ungrouped analysis (Fig. 4 (III)). In contrast to this, Zinc supplementation in healthy participants (9 interventions, n = 730) demonstrated a smaller and insignificant increase in LDL-c (+1.12 mg/dl [95 % CI: −3.93, 6.16; p > 0.05], [I^2^ = 0, p > 0.05]) (Fig. 5 (III)).

### Effect of Zinc supplementation on Triglycerides

Effect of Zinc supplementation on Triglyceride concentration was studied in 19 studies (25 interventions, n = 1,503)[17–23, 26–28, 33–35, 39–43, 46] included in meta analysis. There was a statistically significant reduction in triglyceride concentration in Zinc supplemented group. The pooled mean difference for triglyceride between Zinc supplemented and placebo groups from random effects analysis was −10.92 mg/dl (95 % CI: −18.56, − 3.28; p < 0.01) in the presence of statistical heterogeneity of the data as indicated by I^2^ = 69 % (p < 0.0001) (Fig. 2(IV)). Also sub-analysis of the group of interventions in which Zinc was supplemented alone (21 interventions, n = 1,317) demonstrated statistically significant reduction in TG levels in Zinc supplemented groups in comparison to their controls (−8.73 mg/dl, 95 % CI: −16.29,-1.17, p < 0.05) and statistical heterogeneity as indicated by I^2^ = 64 % (p < 0.0001) (Fig. 3 (IV)). As Fig. 4 (IV) illustrates Zinc supplementation in non-healthy participants (16 interventions, n = 773) demonstrated a significant reduction in TG levels which was greater in magnitude than that in ungrouped analysis (−17.59 mg/dl [95 % CI: −28.80, −6.39; p < 0.05], [I^2^ = 77, p < 0.05]). However, Zinc supplementation in healthy participants (9 interventions, n = 730) did not demonstrate a significant reduction in TG levels (−2.97 mg/dl [95 % CI: −9.75, 3.81; p > 0.05], [I^2^ = 0, p > 0.05]) (Fig. 5 (IV)).

### Other significant effects

Gunasekara, et al. reported a significant reduction in Total cholesterol/HDL ratio from 3.39 to 3.21 (p < 0.05) after Zinc supplementation [46]. Although not statistically significant, a study carried out by Brewer, et al. also reported reduction of this ratio after Zn supplementation in newly diagnosed female patients with Wilson’s disease and patients who had received anti copper therapy (both genders)[49]. Zinc supplementation has shown significant reduction in VLDL cholesterol concentration in few studies [19, 36]. Studies have shown Zinc supplementation results in cholesterol to shift from HDL_3_ to HDL_2_ causing an increase in HDL_2_/HDL_3_ ratio [51, 54].

## Discussion

This comprehensive systematic review and meta-analysis summarize the data from 32 studies involving a total of 14,515 participants. The results of the meta-analysis shows Zinc supplementation alone causes a significant reduction in LDL-c concentration (−4.78 mg/dl, p < 0.05) in the absence of a significant heterogeneity among the studies. Although there is considerable heterogeneity amongst the studies, Zinc supplementation alone reported a statistical significant reduction in serum TC level (−10.72 mg/dl, p < 0.05, I2 = 80 %) and TG level (−8.73 mg/dl, p < 0.05, I2 = 64 %). When analyzed by health status, Zinc supplementation reported a significant reduction in TC, LDL-c and TG levels in non-healthy patients and the magnitude of reduction was greater than that in overall analysis. In healthy patients there was a minor but significant reduction in TC level whereas changes in LDL-c and TG were not significant. When consider HDL-c, Zinc supplementation demonstrated a statistically insignificant increase in the ungrouped analysis whereas a statistically significant increase (+6.15 mg/dl) among non-healthy patients. These findings are in contrast to results from a previous meta-analysis of randomized controlled trials, where no beneficial effects of Zinc supplementation were observed on plasma TC, LDL-c or TG concentrations in overall, ungrouped analysis or when interventions were grouped by health status [25]. Zinc supplementation has demonstrated a significant increase in HDL-c levels among patients with diabetes mellitus in previous meta-analyses which also supports our finding [25, 56]. A finding probably resulting from favorable results demonstrated in several studies reported since the time of the previous meta-analysis [17, 21, 28, 33, 42, 46].

Cardiovascular disease is the leading cause of death in much of the modern world and two major underlying causes are disorders of lipid metabolism and metabolic syndrome [57]. Dyslipidemia is the most important risk factor for atherosclerosis [58]. Atherosclerosis is the main aetiological factor behind coronary artery disease, cerebral vascular disease, and peripheral vascular disease [59]. Within the past decade, clinical trials have demonstrated that LDL-c reduction reduce the clinical cardiac events and the arteriographic investigations have demonstrated that LDL-c reduction can significantly reduce the rate of arteriographically defined disease progression [60]. A recent systematic review and meta-regression analysis concluded that simply increasing the level of circulating HDL-c does not reduce the risk of coronary heart disease events, coronary heart disease deaths, or total death and results supported reduction in low density lipoprotein cholesterol as the primary goal for lipid modifying interventions [61]. Each 40 mg/dl reduction in LDL-c concentration corresponds to 24 % reduction in major cardiovascular events [62]. Therefore, current meta-analysis demonstrates that Zinc supplementation alone can reduce major cardiovascular events by ~2.9 % by lowering LDL-c concentration by 4.78 mg/dl and by 6.8 % in non-healthy individuals by lowering LDL-c by 11.25 mg/dl at an average dose of ~40 mg/day. However, atorvastatin a well established drug for hyperlipidaemia has demonstrated 1.8 mmol/l (69.6 mg/dl) reduction in LDL-c levels at a dose of 10 mg/day in a meta-analysis involving 164 trials [63].

Previous meta-analyses have reported elevated fasting and non-fasting concentrations of TGs were associated with increased risk of coronary heart disease, even after adjustment for HDL-c concentrations [64, 65]. Furthermore three studies between 2007 and 2008 suggested that raised non-fasting TG was strongly associated with increasing risk of myocardial infarction, ischaemic heart disease, ischaemic stroke and all-cause mortality [66–68]. At mild-to-moderately raised triglyceride concentrations (2–10 mmol/l), lipoproteins are small enough to enter into arterial wall and thus have the potential to enter into arterial wall and accumulate causing atherosclerosis [69, 70]. High TG concentrations are a marker for raised remnants rich in cholesterol, which can enter into intima and lead to foam cell formation, atherosclerotic plaques and ultimately cardiovascular disease and increased mortality [71]. Understanding from genetic studies and negative results from randomized trials is low HDL-c might not cause cardiovascular disease as originally thought and this understanding has now generated an interest in elevated levels of TGs [71]. Therefore Zinc supplementation could reduce the cardiovascular events and deaths, as it results in significant reduction in TC, TG and LDL-c.

Dyslipidemia is one of the major risk factors for cardiovascular disease in diabetes mellitus. High plasma TG, increased small dense LDL-c particles, low HDL-c are characteristic features of diabetic dyslipidemia and these lipid changes are mainly attributed to increased free fatty acid flux secondary to insulin resistance [72]. The increase in cardiovascular risk in obesity depends to a significant extent on changes in lipid profile, mainly decreased HDL-c and increased TG and insulin resistance is the central cause for these changes [73]. Prominent and known risk factors that contribute to the increased incidence of atherosclerosis in hemodialysis patients are disorders in lipoprotein metabolism and elevated plasma fibrinogen concentrations [74]. Therefore the participants we categorized as non-healthy (patients with - type 2 diabetes, End stage renal failure and on haemodialysis and obesity) are at increased risk of dyslipidemias. Zinc supplementation significantly reduces TC, LDL-c and TG and elevates HDL-c in non-healthy patients. Elevated plasma concentrations of HDL-c are associated with protection from atherosclerotic cardiovascular disease. Cardio protective effect of HDL-c is due to its role in reverse cholesterol transport in which cholesterol from peripheral tissues is returned to the liver for excretion in the bile, its protective effect on endothelial cells and its antioxidant activity [75]. All these evidence support that Zinc supplementation will effectively reduce the cardiovascular risk among non-healthy patients.

Our results demonstrated HDL-c concentration was significantly reduced due to Zinc supplementation among healthy participants. A previous meta-analysis also showed that Zinc supplementation among healthy individuals was associated with a significant reduction in HDL-c concentration supporting our finding [25]. Low HDL-c (=/< 40 mg/dl) is one of the 5 major Coronary Heart Disease (CHD) risk factors, and HDL-c level is also a component of the Framingham scoring system, the method used to estimate 10-year CHD risk and determine the intensity of lipid-lowering therapy [75]. Furthermore Zinc supplementation did not demonstrate a significant reduction in LDL-c or in TG despite a minor reduction in TC. Therefore Zinc supplementation may not have much beneficial effects in healthy people.

Several molecular mechanisms are believed to be involved in reduction in serum lipid levels following Zinc supplementation. In Zinc-deficient rats lowered plasma HDL-c and some apoproteins (A1, A2, C and E) but also elevated total cholesterol concentrations have been observed [76, 77]. On the other hand, Zinc supplementation has been shown to inhibit the development of atherosclerosis in rabbits fed a high cholesterol diet [78]. It is well documented that Zinc is an important mediator of insulin storage and secretion from the pancreas [78]. In addition, pancreatic beta-cells utilize a very efficient transporter (ZnT8) to accumulate Zinc inside the cells. Thus, Zinc deficiency or alterations in ZnT8 expression have a potential to depress insulin secretion [79]. Zinc enhances the phosphorylation of insulin-receptor substrates to activate a series of signal transduction, improving insulin sensitivity [80, 81]. Insulin resistance at the adipocytes results in increased release of fatty acids into the circulation and then increased free fatty acid flux to the liver stimulates the assembly and secretion of VLDL resulting in hypertriglyceridemia [82]. Zinc supplementation either improving insulin secretion or reducing insulin resistance as described above inhibits the lipolysis in adipose tissues, reduce free fatty acid release into the circulation and its availability to the liver and excessive lipoprotein synthesis. Besides Zinc contribution to insulin secretion and action, Zinc directly affects lipid metabolism. Recently it has been shown that Zinc deficiency down regulates fatty acid utilization in mitochondria and peroxisomes and up regulates lipid synthesis in the rat liver affecting the expression of genes encoding enzymes contributing to liver lipid homeostasis [83].

The present meta-analysis has notable strengths. These include 1) large number of individuals in the sub group analysis in which the effect of Zinc alone supplementation was studied (n = 1,528), 2) studies were assessed using jaded scale score and the studies with poor methodological quality were excluded from meta-analysis, 3) use of random effect model of meta-analysis which allow heterogeneity among studies and 4) average dose of elemental Zinc used in the interventions included in meta-analysis (39.3 mg/d) does not exceed the tolerable upper intake level (40 mg elemental Zinc per day in adults) [84]. A limitation of present meta analysis was presence of considerale heterogeneity when assessing the effect of Zinc supplementation on TC anc TG concentrations which stems from; a) Variations in baseline parameters such as serum Zinc status and lipid levels, b) Differences in Zinc doses, formulae, sample sizes and study durations, and c) Limited availability of data on Zinc intake from other sources such as diet.

## Conclusions

The present meta-analysis demonstrates that Zinc supplementation has favourable effects on plasma lipid parameters. Zinc supplementation significantly reduced total cholesterol, LDL cholesterol and triglycerides. In addition to that, Zinc supplementation in non-healthy patients demonstrated a significant elevation of HDL cholesterol. Therefore it may have the potential to reduce the incidence of atherosclerosis related morbidity and mortality especially in non-healthy patients who are at risk of atherosclerosis.

## Abbreviations

FBG: Fasting blood glucose
HbA1c: Glycosylated hemoglobin
HDL-c: High density lipoprotein cholesterol
LDL-c: Low density lipoprotein cholesterol
TG: Triglycerides
TC: total cholesterol
RCT: Randomized control trial
MVM: Multi vitamin mineral
